# Promoting cognitive health through the nexus of gut microbiota and dietary phytochemicals

**DOI:** 10.3389/fnut.2025.1636131

**Published:** 2025-08-29

**Authors:** Lin Luo

**Affiliations:** ^1^School of Physical Education, Guizhou Normal University, Guiyang, China; ^2^Key Laboratory of Brain Function and Brain Disease Prevention and Treatment of Guizhou Province, Guiyang, China

**Keywords:** gut microbiota, phytochemicals, cognitive function, gut-brain axis, urolithins, sulforaphane, equol, hesperidin

## Abstract

The intricate interactions between gut microbiota and cognitive function have become a forefront topic at the convergence of neuroscience and nutrition. This review systematically evaluates the bidirectional relationship between dietary phytochemicals and gut microbiota, highlighting their potential mechanisms for promoting cognitive health. The review begins by describing how gut microbiota dysbiosis can contribute to cognitive decline by transmitting gut-derived signals to the central nervous system via the gut–brain axis. Subsequently, the discussion focuses on how phytochemicals act as modulators of gut microbiota composition and undergo microbial-mediated metabolic transformation. Special attention is paid to four key microbial-derived metabolites—urolithins, sulforaphane, equol, and hesperidin—that exhibit neuroprotective effects through antioxidative, anti-inflammatory, neuroprotective, and metabolic regulatory pathways. Furthermore, the review examines how individual variability in gut microbiota composition influences the efficiency of phytochemical biotransformation and underscores the implications for precision nutrition interventions. Emerging evidence indicates that the synergistic regulation of the gut–brain axis by dietary phytochemicals and gut microbiota offers a robust theoretical basis for developing novel strategies to preserve cognitive function. Future research should further clarify the molecular mechanisms underlying specific microbe–phytochemical interactions and accelerate the clinical translation of personalized nutrition strategies.

## 1 Introduction: the complex interplay between diet, gut microbiota, and cognition

**Figure 1:**
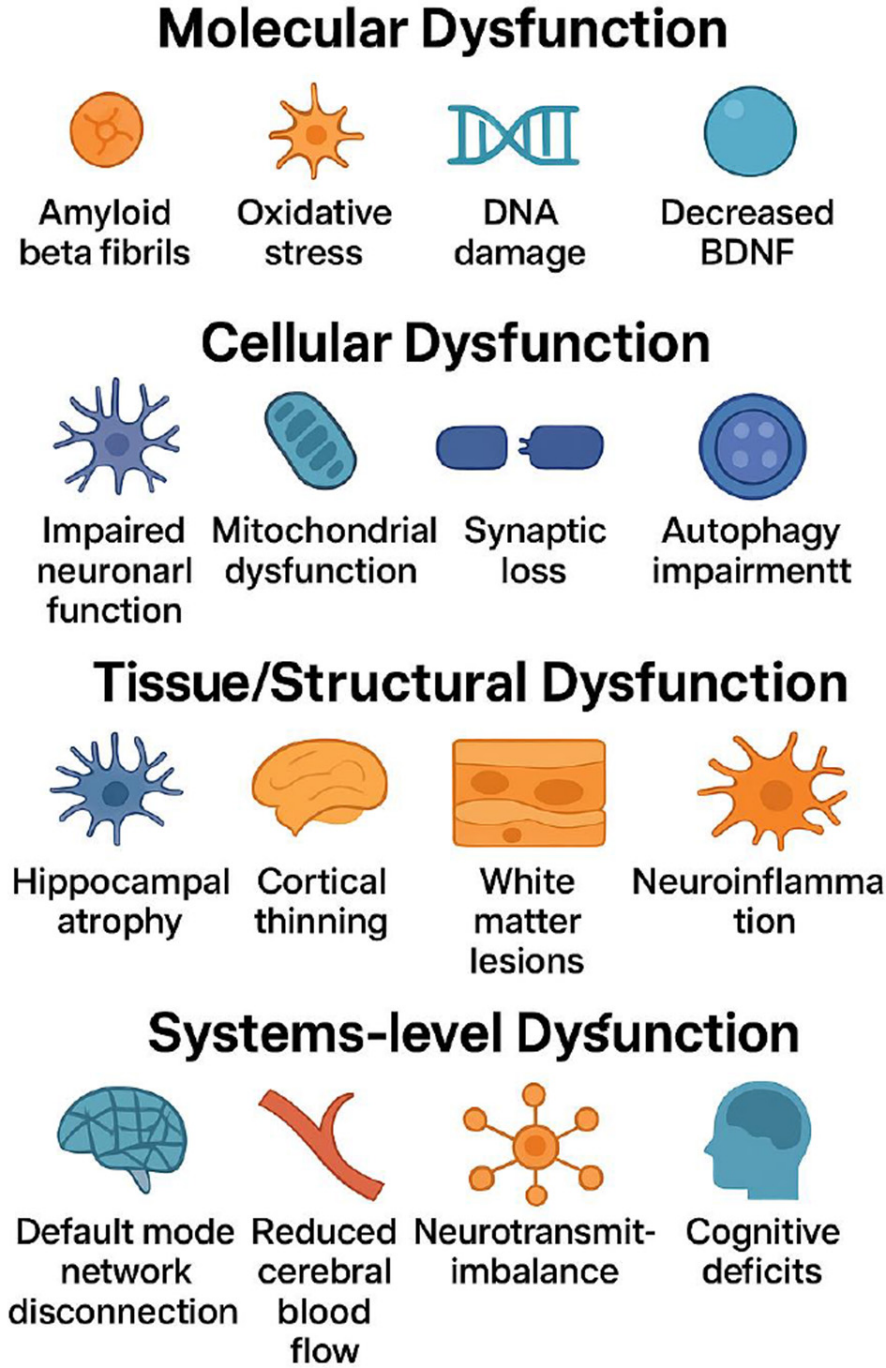
Multilevel pathological mechanisms of progressive cognitive decline: from molecular to cellular, tissue, and systems levels (The process initiates at the molecular level with amyloid-β fibril formation, oxidative stress, DNA damage, and reduced BDNF levels that trigger cellular injury. These molecular disruptions progress to cellular dysfunction, characterized by impaired neuronal function, mitochondrial failure, synaptic loss, and autophagy impairment that compromise fundamental neural processes. Continued damage leads to tissue/structural dysfunction, including hippocampal atrophy, cortical thinning, white-matter lesions, and neuroinflammation with blood-brain barrier disruption, reflecting macroscopic brain pathology. The cascade culminates in system-level dysfunction, where large-scale network disconnection (e.g., default mode network), reduced cerebral blood flow, neurotransmitter imbalances, and manifest cognitive deficits represent clinical endpoints.).

Progressive cognitive decline is a hierarchical pathological process characterized by gradual alterations at the molecular, cellular, and systemic levels (Figure 1). Ultimately, these changes manifest as impaired bodily function and reduced overall health status (1–3). Clinically, affected individuals frequently present with memory impairment, deficits in attention and executive function, diminished language and information processing abilities, as well as a spectrum of neuropsychiatric symptoms. Collectively, these deficits undermine independence in activities of daily living (4–6).

In the past decade, extensive research has identified the gut microbiota as a central regulator of brain aging and cognitive function (7). Disruptions in the composition, diversity, or functionality of the microbiota—referred to as “dysbiosis”—have been strongly linked to adverse cognitive outcomes (8, 9). Beyond taxonomic alterations, dysbiosis also reshapes the spectrum of microbiota-derived metabolites. These metabolites influence brain health by modulating neuroinflammation, synaptic plasticity, and mitochondrial function (10–12). Notably, older adults who retain a “youthful” microbial profile tend to show better-preserved cognitive function and produce a greater diversity of beneficial metabolites (11, 13), highlighting the substantial potential for targeting the gut ecosystem in cognitive health interventions.

Diet is the most influential and modifiable external factor in shaping the composition and function of the gut microbiota. Systematic reviews have shown that dietary patterns rich in whole grains, fruits, vegetables, and other fiber-dense plant-based foods—including, but not limited to, the Mediterranean and DASH diets—while minimizing their intake of refined sugars and saturated fats, are associated with a significantly lower risk of developing neurodegenerative diseases such as Alzheimer's disease and Parkinson's disease (14, 15). A common characteristic of these dietary patterns is their abundance of phytochemicals—diverse, non-nutritive secondary metabolites derived from plants. Phytochemicals exert their effects both directly on the host and indirectly via microbiota-mediated biotransformation, producing bioactive metabolites that act along the gut–brain axis (16).

Polyphenols are a representative example: the gut microbiota can degrade their complex structures into smaller, highly bioavailable derivatives, thereby enhancing the integrity of the intestinal barrier and mitigating neuroinflammation and oxidative stress (17). Similarly, phytochemicals such as curcumin and resveratrol modulate the gut microbial ecosystem by increasing beneficial taxa and suppressing opportunistic pathogens, together creating a neuroprotective environment that supports brain homeostasis (18, 19).

This review aims to comprehensively synthesize the bidirectional communication between dietary phytochemicals and the gut microbiota, elucidating the molecular mechanisms by which these interactions support cognitive health. Specifically, we address the following key questions:

How does the gut microbiota regulate cognitive function through the gut–brain axis?

In what ways do phytochemicals reprogram the composition and metabolic activity of the microbiota?

Through which pathways do microbiota-derived phytochemical metabolites exert neuroprotective effects?

Addressing these questions will not only deepen our understanding of the biological foundations of cognitive decline but also provide a theoretical framework for the development of precision nutrition and microbiota-targeted strategies to prevent or delay cognitive impairment.

## 2 Gut microbiota and cognitive function

### 2.1 The role of gut microbiota in health and cognition

As the largest and most intricate microbial ecosystem in the human body, the gut microbiota is composed of approximately 3.8 × 10^13^ microorganisms. Its composition and function are shaped by a multitude of factors—including host genetics, dietary patterns, lifestyle, and disease status—resulting in pronounced inter-individual heterogeneity and dynamic shifts across the human lifespan (20, 21). Through the neuroendocrine, immune, and metabolic axes of the gut–brain axis, the gut microbiota establishes a multi-layered, bidirectional communication network with the central nervous system. This network exerts profound effects on brain development, information processing, and the aging process (22, 23).

#### 2.1.1 Metabolic signaling: SCFAs and the blood–brain barrier

From a metabolic perspective, short-chain fatty acids (SCFAs)—including acetate, propionate, and butyrate—serve as key signaling molecules bridging the gut and the brain. Butyrate-producing bacteria, notably from the phylum Firmicutes, ferment dietary fiber to generate these metabolites (24). SCFAs contribute to central nervous system health by activating G protein-coupled receptors (GPR41/43), inhibiting histone deacetylase (HDAC) activity, and upregulating tight junction proteins. These processes reinforce blood–brain barrier (BBB) integrity, regulate microglial maturation, and suppress lipopolysaccharide (LPS)-mediated neuroinflammation, ultimately supporting hippocampus-dependent memory and spatial navigation (25, 26). Importantly, lower plasma SCFA levels have been strongly associated with reduced cognitive scores in patients with Alzheimer's disease (AD), supporting their potential as both biomarkers and intervention targets (27, 28).

#### 2.1.2 Neurotransmitter modulation: serotonin and GABA

In addition to metabolic signaling, the gut microbiota plays a pivotal role in neurotransmitter synthesis and regulation. Approximately 95% of serotonin (5-hydroxytryptamine, 5-HT) is synthesized in intestinal enterochromaffin cells, a process that is dependent on aromatic amino acid decarboxylase signals secreted by certain Bacillus species (29). Gut-derived 5-HT influences vagal nerve activity, platelet serotonin release, and energy metabolism, thereby indirectly impacting mood and cognition. Its precursor, 5-hydroxytryptophan (5-HTP), can cross the BBB and be converted to 5-HT in the brain, directly promoting synaptic plasticity (30, 31).

Moreover, the gut microbiota sustains cerebral chemical homeostasis by regulating the supply of neurotransmitter precursors. Certain strains of Lactobacillus are capable of producing γ-aminobutyric acid (GABA), while Bifidobacterium species enhance glutamate decarboxylase activity to accelerate GABA synthesis. Additionally, shifts in microbial metabolism of indole derivatives and disturbances in the tryptophan–kynurenine pathway have been strongly associated with cognitive impairments resembling depressive phenotypes (32, 33).

#### 2.1.3 Bile acid signaling and the gut–brain axis

Bile acid metabolism represents another critical route within the gut–brain axis. Gut microbes facilitate the transformation of primary bile acids into secondary bile acids via 7α-dehydroxylation. These secondary metabolites interact with the farnesoid X receptor (FXR) and Takeda G protein-coupled receptor 5 (TGR5), modulating neuroinflammation, mitochondrial function, and BDNF–CREB signaling, thereby contributing to memory consolidation and emotional stability (34, 35). Recent animal studies further demonstrate that interactions among gut microbiota, bile acids, and TGR5 receptors significantly influence host metabolism and intestinal barrier function, suggesting novel avenues for therapeutic intervention (36).

#### 2.1.4 Clinical and translational evidence

Collectively, the gut microbiota shapes central nervous system function and cognitive performance through multiple, interconnected pathways—including SCFA–BBB integrity, 5-HT–neuroplasticity, GABA–synaptic inhibition, and secondary bile acid–receptor signaling.

However, there are inconsistencies across clinical and animal studies regarding specific microbial taxa–function relationships. For example, some studies have observed reduced abundance of beneficial genera such as Lactobacillus and Bifidobacterium and increased levels of potential pathogens in patients with AD and mild cognitive impairment (MCI) (37, 38). Conversely, a recent meta-analysis of ten randomized controlled trials (*n* = 419) found no significant improvement in total Mini-Mental State Examination (MMSE) or Montreal Cognitive Assessment (MoCA) scores after 8–24 weeks of probiotic supplementation (39). These discrepancies may be attributable to heterogeneity in disease stage, the probiotic strains or combinations used, dosage, and duration of follow-up—highlighting the need for personalized approaches to probiotic interventions.

Before a robust causal chain linking specific microbiota changes, metabolic signaling, and cognitive outcomes can be established, more rigorous and standardized multi-omics studies are essential to improve evidence quality and enhance clinical translatability.

### 2.2 Interactions among dysbiosis, cognitive decline, and inflammation

Dysbiosis refers to a disrupted state of gut microbial composition and function and is closely linked to both cognitive decline and chronic inflammation (40, 41). The underlying mechanisms involve several key pathways.

#### 2.2.1 Intestinal barrier disruption and systemic inflammation

Under homeostatic conditions, short-chain fatty acids (SCFAs) induce the secretion of MUC2 and maintain the expression of tight junction proteins such as ZO-1 and Claudin-1, thereby preserving intestinal barrier integrity (42). When microbial abundance and metabolic profiles are altered, mucin-producing bacteria (e.g., mucinase-positive Bacteroides) decrease, while pro-inflammatory Gram-negative bacteria increase, resulting in greater barrier permeability. Consequently, exogenous lipopolysaccharides (LPS) enter the circulation, activating the TLR4–MyD88–NF-κB signaling cascade and inducing the production of pro-inflammatory cytokines such as IL-1β, IL-6, and TNF-α (43). These cytokines can cross the blood–brain barrier (BBB) and activate microglia, leading to dendritic spine retraction, reduced long-term potentiation (LTP), and ultimately, impairments in learning and memory (44). Recent *in vivo* studies have further confirmed that gut-derived LPS from the Enterobacteriaceae family significantly upregulates hippocampal TNF-α mRNA in mice within 48 h and prolongs escape latency in the Morris water maze test (45).

#### 2.2.2 Neurotransmitter metabolic disruption—The serotonin pathway

Dysbiosis reduces the production of metabolites such as butyrate, thereby inhibiting intestinal tryptophan hydroxylase 1 (TPH1) activity and impeding peripheral serotonin (5-HT) synthesis (46). Simultaneously, a pro-inflammatory environment diverts tryptophan metabolism toward the kynurenine pathway, resulting in the accumulation of neurotoxic metabolites such as quinolinic acid and 3-hydroxykynurenine (47). This dual effect not only depletes serotonin precursors but also suppresses the synthesis of GABA and acetylcholine, weakening synaptic plasticity and emotional regulation (48, 49). In human studies, a lower plasma 5-HTP/kynurenine ratio is significantly correlated with reduced MoCA scores, underscoring the mediating role of the metabolic axis in cognitive decline (50).

#### 2.2.3 Mitochondrial dysfunction and oxidative stress

Microbial-derived metabolites, such as indole-3-propionic acid, activate the PGC-1α-NRF1 pathway and promote neuronal mitochondrial biogenesis. Under dysbiotic conditions, these protective signals are diminished, resulting in reduced ATP production and increased reactive oxygen species (ROS) accumulation—creating a vicious cycle of mitochondrial injury and oxidative stress (51, 52). Recent multi-omics studies have shown that patients with AD exhibit reduced fecal levels of butyrate-producing bacteria such as Butyrivibrio, which correlates closely with lower plasma mitochondrial DNA copy number and poorer verbal fluency (53). In neonatal hypoxia-ischemia models, antibiotic-induced dysbiosis significantly amplifies hippocampal ROS levels and worsens long-term cognitive deficits (54).

Although animal models and some open-label clinical studies consistently suggest that modulation of gut microbiota through probiotics, prebiotics, or fecal microbiota transplantation (FMT) can reduce inflammatory burden and confer cognitive benefits (55), high-quality randomized controlled trials (RCTs) have yet to yield consistent conclusions. A recent systematic review and meta-analysis including 10 RCTs (*n* = 778) reported an overall standardized mean difference (SMD) of ≈0.52 for probiotics on global cognition, albeit with substantial heterogeneity (*I*^2^ = 68%) (56). Conversely, another meta-analysis focusing on populations with mild cognitive impairment and Alzheimer's disease (*n* = 419) observed no significant improvement in MMSE or MoCA scores, indicating that intervention duration, strain composition, and disease stage may be decisive factors for efficacy (39). Regarding interventions, the first multicenter, double-blind phase II FMT trial (GUT-PARFECT) significantly improved motor symptoms in Parkinson's disease patients but failed to yield statistically significant benefits on cognitive scales (57).

Overall, current research remains limited by sample sizes generally < 100, follow-up periods of ≤ 12 weeks, a lack of uniform cognitive assessment tools and time points, and insufficient integration of metagenomic, metabolomic, and neuroimaging data. These methodological shortcomings hinder the construction of a clear “microbe–metabolite–brain phenotype” causal chain (58). Such limitations help explain discrepancies in clinical evidence and highlight the urgent need for multi-omics tracking and long-term randomized trial designs in future research.

## 3 Key mechanisms linking dietary phytochemicals and cognitive function

### 3.1 Classification, sources, and bioactivities of phytochemicals

Phytochemicals are non-essential, bioactive trace compounds widely found in plant-based foods. To date, over 100,000 types have been identified, with the major groups including polyphenols, carotenoids, isothiocyanates, organosulfur compounds, terpenoids, and phytoestrogens (59). Although these compounds are not essential nutrients for humans, they have attracted significant attention due to their antioxidant, anti-inflammatory, and chronic disease prevention properties. Their fate in the human body is determined by factors such as molecular size, lipophilicity, blood–brain barrier (BBB) permeability, chemical stability, and, crucially, gut microbial metabolism (60, 61). Many parent molecules, due to their polarity or steric hindrance, have difficulty crossing the BBB. However, after transformation by microbial-specific enzymes into sulfate or glucuronide conjugates, these metabolites can be detected in brain tissue and often exhibit enhanced neuroactivity (62, 63). The classification, typical dietary sources, and representative bioactivities of major dietary phytochemicals are summarized in Table 1.

**Table 1:** Major dietary phytochemicals: classification, food sources, and bioactivities.

**Category**	**Representative compounds**	**Main food sources**	**Bioactivity**
Polyphenols (130, 131)	Anthocyanins, flavanols, catechins	Berries, grapes, tea, cocoa	Antioxidant, anti-inflammatory, neuroprotective
Isothiocyanates (132, 133)	Sulforaphane, indole-3-carbinol	Broccoli, cabbage, kale	Antioxidant, anti-inflammatory, detoxification
Carotenoids (134, 135)	Beta-carotene, lycopene	Carrots, tomatoes, pumpkins	Antioxidant, retinal protection
Organosulfur Compounds (135, 136)	Allicin, diallyl sulfides	Garlic, onions, leeks	Antioxidant, antimicrobial, antithrombotic
Terpenoids (137, 138)	Curcumin, carvacrol	Turmeric, rosemary, thyme	Anti-inflammatory, neuroprotective
Phytoestrogens (139, 140)	Soy isoflavones, lignans	Soybeans, flaxseeds, whole grains	Estrogen-like effects, antioxidant

Current mechanistic studies mainly focus on: (1) scavenging free radicals and activating the Nrf2–ARE antioxidant pathway (64), (2) inhibiting the NF-κB–MAPK inflammatory cascade (65), (3) modulating neurotransmitter and neurotrophic factor levels (66), and (4) epigenetic reprogramming via histone acetylation and DNA methylation (66). However, systematic comparisons of the pharmacokinetics, dose–response relationships, and BBB permeability among different classes of phytochemicals remain limited (60, 67, 68). This gap has partially constrained the extrapolation of results and their clinical translation.

### 3.2 The phytochemical–gut microbiota–brain axis: a prebiotic perspective and current evidence

**Figure 2:**
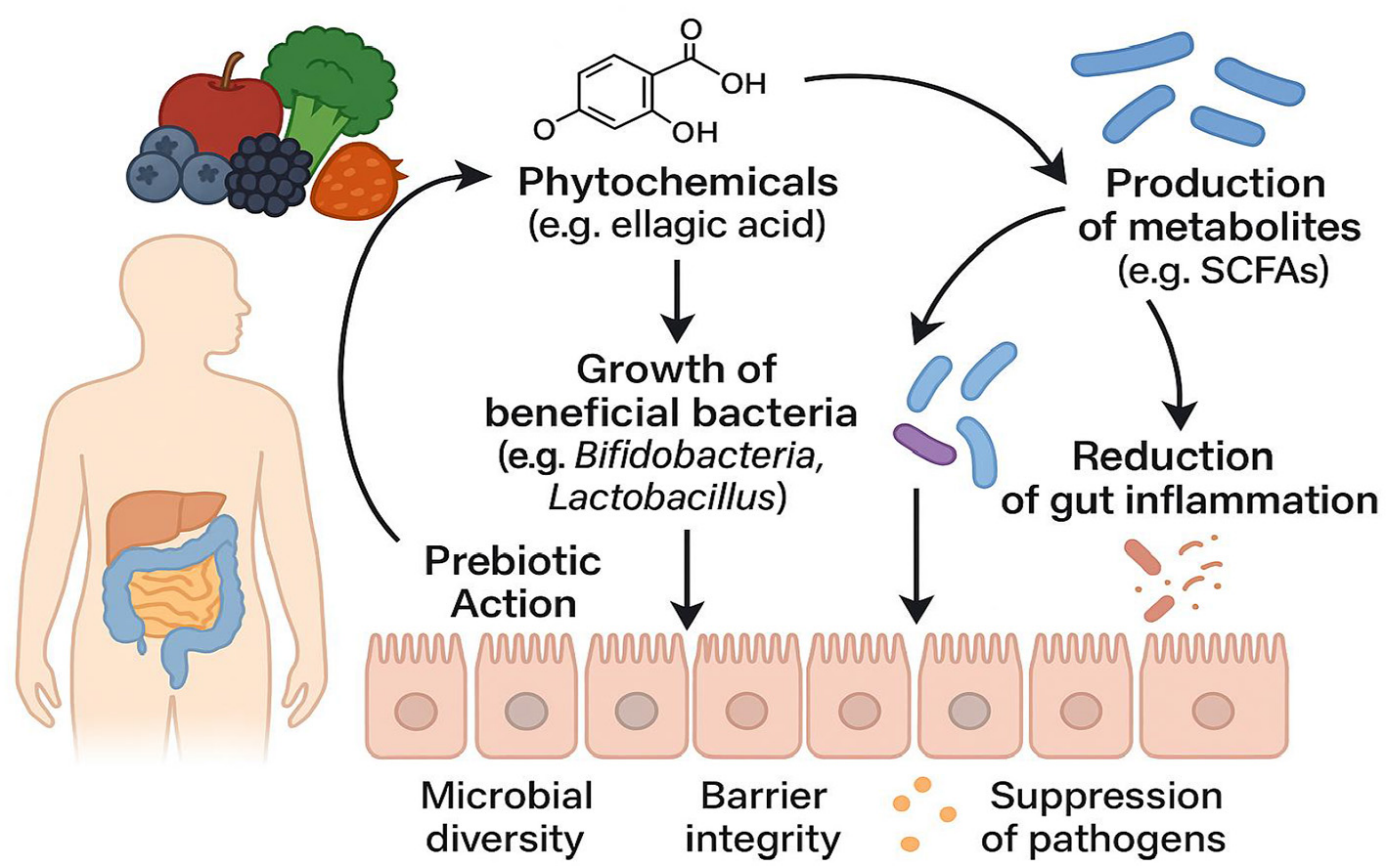
Schematic Illustration of Prebiotic Mechanisms in Phytochemical–Gut Microbiota Interactions (Dietary phytochemicals, such as ellagic acid and related compounds, exert prebiotic effects upon reaching the colon through three principal pathways: (1) to selectively promote the proliferation of beneficial bacteria—such as Bifidobacterium and Lactobacillus—thus enhancing microbial diversity, (2) to enhance the metabolism of beneficial microbes and stimulate the production of functional metabolites (e.g., short-chain fatty acids, SCFAs), which in turn suppress potential pathogens and reduce intestinal inflammation, and (3) to comprehensively strengthen intestinal barrier integrity.).

Early research primarily attributed the neuroprotective effects of phytochemicals to their direct antioxidant and anti-inflammatory actions. However, pharmacokinetic data demonstrate that most parent phytochemical molecules exist in plasma and cerebrospinal fluid at extremely low free concentrations, largely in conjugated forms. This indicates that the true “active agents” mediating neuroprotection are likely microbiota-derived metabolites (63). From a prebiotic perspective—defined here as the ability of dietary components to selectively promote the growth and metabolic activity of beneficial gut microbes—phytochemicals serve dual roles: they act as “metabolic substrates” for the microbiota and simultaneously “reshape the microbial ecosystem” by selectively inhibiting or promoting specific taxa (16, 69) (see Figure 2).

For example, interventions with berry anthocyanins have been shown in both animal and human studies to increase the abundance of butyrate-producing bacteria and elevate short-chain fatty acid (SCFA) levels, with these changes correlating with improved spatial memory (70). Importantly, phytochemicals may exhibit a “double-edged sword” effect—meaning their benefits and risks depend on dose and duration. For instance, oral curcumin has been shown to ameliorate memory deficits in APPswe/PS1dE9 mice by inhibiting the HMGB1–RAGE/TLR4–NF-κB pathway, yet high doses or prolonged use can reduce hepatic and renal iron content and adversely affect reproductive function (71–73).

Recent evidence from a meta-analysis of 24 trials (*N* = 2,336) indicates that long-term polyphenol supplementation in adults aged ≥60 produces only mild improvements in immediate recall, with no significant effects on delayed recall or executive function and considerable between-study heterogeneity (*I*^2^ > 60%) (74). Similarly, systematic reviews of curcumin interventions report that only two double-blind RCTs have observed meaningful cognitive benefits in populations with cognitive impairment or dementia; most studies in healthy or metabolically abnormal individuals have shown limited advantage (75). Furthermore, a 12-week matcha intervention in Japanese older adults improved emotion recognition and sleep quality but did not yield statistically significant changes in MMSE or MoCA scores (76).

The mechanistic targets of different phytochemicals vary considerably.

Polyphenols primarily enhance synaptic plasticity and antioxidative defenses (77); isothiocyanates are more associated with detoxification and anti-inflammatory pathways (78); and terpenoids (e.g., curcumin) exhibit both anti-inflammatory and epigenetic regulatory effects (79).

Nonetheless, several key challenges remain: the majority of RCTs have relatively short follow-up periods ( ≤ 16 weeks), highly variable intervention doses, delivery matrices (e.g., food vehicles or supplement forms), and marked differences in baseline microbiota composition. This limits the ability to detect cumulative cognitive effects. There is a lack of integrated multi-omics approaches; only a few studies concurrently assess metagenomic and metabolomic changes, making it difficult to establish a robust causal “phytochemical–microbiota metabolism–central phenotype” chain. Variability in individual microbiome backgrounds further complicates efficacy assessment and clinical translation.

Collectively, current evidence suggests that the cognitive benefits of phytochemicals are largely dependent on their biotransformation into low-molecular-weight metabolites by the gut microbiota and the consequent remodeling of microbial community structure. However, the effectiveness of these interventions is limited by factors such as dose, intervention duration, and inter-individual microbiome variability. There is a clear need for long-term, stratified, multi-omics randomized trials to elucidate optimal compound–microbiota matching strategies for cognitive health and advance the clinical translation of precision diet–microbiota interventions, thereby capturing the full complexity of the diet–microbiota–brain axis.

### 3.3 Molecular mechanisms of microbiota-derived metabolic pathways

#### 3.3.1 Short-chain fatty acid (SCFA) pathway

Butyrate enhances long-term potentiation (LTP) and synaptic plasticity by activating GPR41/43 receptors and inhibiting histone deacetylase (HDAC) activity. These actions result in the downregulation of inflammatory mediators such as IL-1β and TNF-α and the upregulation of hippocampal BDNF expression, ultimately supporting cognitive function and reducing neuroinflammation (9, 80). Notably, in D-galactose-induced accelerated aging models, intervention with citrus flavonoids and hawthorn polysaccharides significantly increases fecal butyrate levels and reverses spatial memory deficits. This highlights a mechanistic chain: polyphenols or polysaccharides, through butyrate-producing bacteria, increase butyrate production, which in turn upregulates BDNF (81). However, most human intervention studies have small sample sizes, short durations, and often lack adequate control for dietary fiber intake in the control group. These limitations may overestimate the true contribution of SCFAs to cognitive improvement (82, 83).

#### 3.3.2 Bile acid signaling pathway

Metabolomic studies reveal that patients with Alzheimer's disease (AD) show a shift characterized by reduced primary bile acids (PBAs) and increased neurotoxic secondary bile acids (SBAs). The severity of this imbalance is negatively correlated with MMSE scores, indicating cognitive decline (84). Animal experiments demonstrate that curcumin can upregulate PBAs and suppress SBAs via the FXR–SHP axis, thereby improving spatial learning and memory (85, 86). Despite these promising findings, clinical RCTs targeting bile acid modulation remain very limited. Furthermore, the strain-specific and dose–response relationships of Ruminococcus and Eubacterium in bile acid transformation have not yet been validated in human populations, limiting the translation to personalized interventions (87, 88). Future research should utilize integrated metagenomic and humanized mouse model approaches to clarify the causal relationship of the microbiota–bile acid–FXR/TGR5–BDNF signaling axis in cognitive health.

#### 3.3.3 Tryptophan–kynurenine metabolic pathway

The gut microbiota determines whether tryptophan is metabolized toward serotonin (5-HT) synthesis or the kynurenine (KYN) branch. Human studies indicate that higher levels of KYN and 3-hydroxykynurenine are negatively correlated with cognitive performance, whereas indole-3-propionate (IPA), which is enriched in centenarians, may exert neuroprotective effects (89). In DSS-induced colitis models, dysbiosis is associated with upregulation of IDO-1/TDO-2 and inhibition of KAT2, resulting in KYN accumulation within the blood–brain barrier and subsequent cognitive impairment (90, 91). These findings underscore that inflammation-driven IDO-1 activation is a key driver of KYN elevation and that the modulation of the microbiota composition–tryptophan metabolic profile may alleviate both gut inflammation and central cognitive disorders, thus representing a promising therapeutic avenue.

Overall, while current evidence suggests that phytochemicals can improve cognition by upstream remodeling of the gut microbiota and its metabolic products, human trials remain limited by small sample sizes, short follow-up periods, and insufficient mechanistic measurements (e.g., metabolite quantification and receptor expression profiling). There is an urgent need for long-term, integrated, and stratified randomized studies to confirm the mediating effects of these pathways across different ages and disease stages and to identify the optimal combinations of compounds, bacterial strains, and dosages for individualized cognitive interventions.

## 4 Microbial-derived metabolites from dietary phytochemicals and their cognitive benefits

Microbial metabolites derived from dietary phytochemicals play a pivotal role in maintaining cognitive health. The gut microbiota possesses a vast and flexible metabolic network capable of enzymatically transforming a wide range of phytochemicals into unique bioactive metabolites. These microbial-derived metabolites typically exhibit greater bioavailability and distinct biological activities, acting through multiple pathways to influence both the gut and central nervous systems.

**Figure 3:**
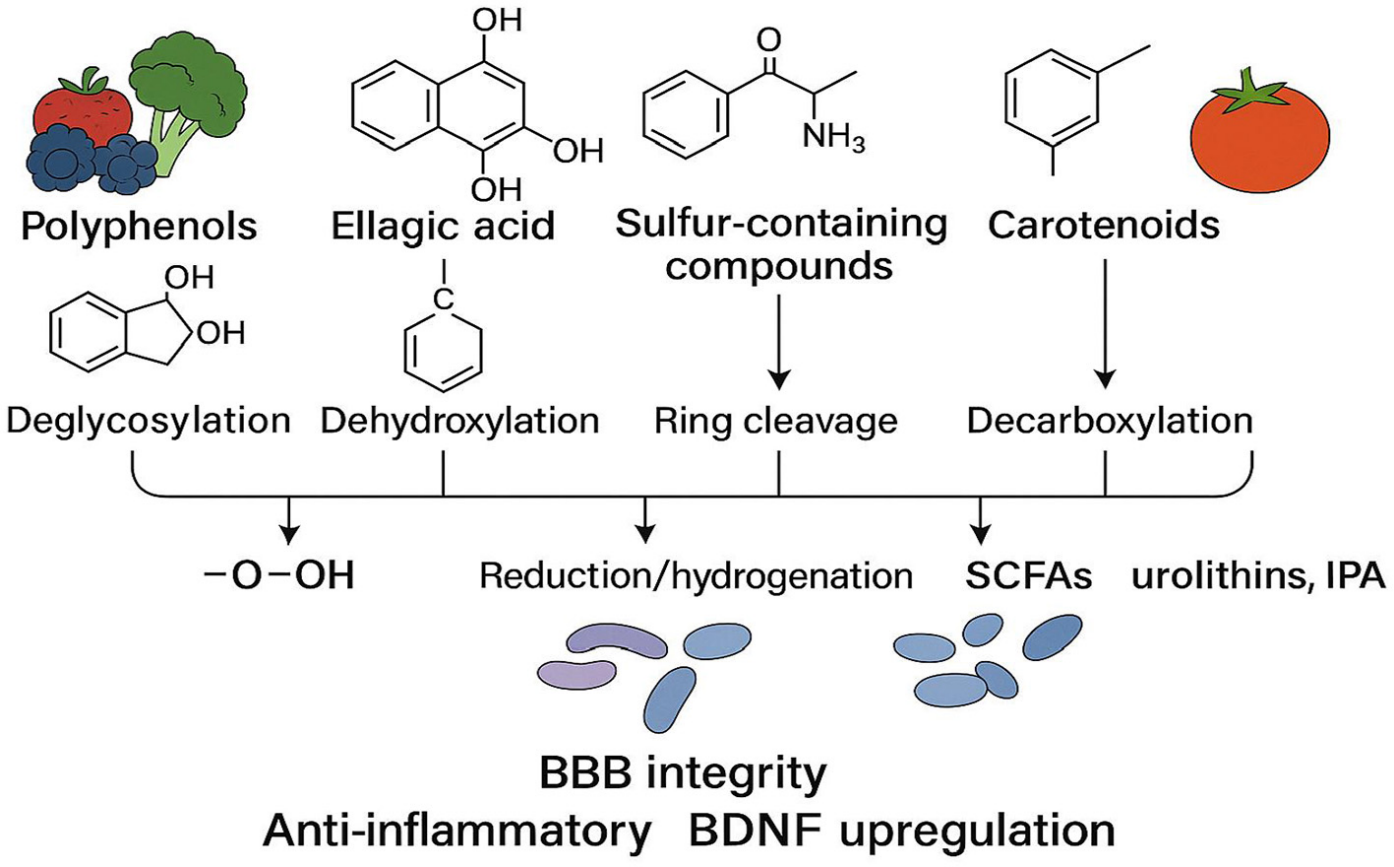
Microbial biotransformation pathways of dietary phytochemicals [Upon catalysis by specific microbial enzyme systems, dietary phytochemicals—ranging from polyphenols, sulfur-containing compounds, nitrogen-containing compounds, to carotenoids (from left to right)—undergo reactions such as deglycosylation, dehydroxylation, ring cleavage, and decarboxylation, resulting in small-molecule metabolites with higher bioavailability. Representative products include short-chain fatty acids (SCFAs), urolithins, and indole-3-propionate (IPA). These metabolites collectively promote synaptic plasticity and neuroprotection by enhancing blood–brain barrier (BBB) integrity, suppressing neuroinflammation, and upregulating brain-derived neurotrophic factor (BDNF).].

As illustrated in Figure 3, the gut microbiota can metabolize polyphenols, sulfur-containing compounds, nitrogen-containing compounds, and carotenoids via processes such as deglycosylation, dehydroxylation, ring cleavage, and decarboxylation, thereby generating small-molecule metabolites (e.g., SCFAs, urolithins, and IPA) with higher bioavailability. These products reinforce BBB integrity, inhibit neuroinflammation, and increase BDNF expression, ultimately supporting cognitive function through the gut–brain axis.

This section focuses on four categories of microbial-derived metabolites from phytochemicals that are especially relevant to cognitive function: urolithins, sulforaphane, equol and hesperetin. We discuss their dietary sources, precursor phytochemicals, the bacterial taxa involved in their production, and their mechanisms of action in supporting cognitive health.

### 4.1 Urolithins: key products of the ellagitannin–ellagic acid pathway

Pomegranate, strawberries, and walnuts are notable dietary sources of ellagitannins. These compounds are hydrolyzed in the upper gastrointestinal tract to release ellagic acid (EA). In the colon, ellagic acid undergoes sequential dehydroxylation, ring opening, and decarboxylation, catalyzed by gut bacteria such as Gordonibacter and Ellagibacter. This microbial transformation leads to the production of urolithin A (Uro-A) and urolithin B (Uro-B) (92, 93). The presence or absence of the ellagic acid decarboxylase gene cluster defines three metabolic phenotypes: UM-0 (non-producers), UM-A (Uro-A predominant), and UM-B (Uro-B and iso-Uro-A predominant), reflecting inter-individual variability in microbial metabolism (94).

Animal studies have shown that oral Uro-A (300 mg·kg^−1^ for 14 days) significantly improves spatial memory, reduces neuronal apoptosis, and promotes neurogenesis in APP/PS1 mice (95). Pharmacokinetic data indicate that Uro-A is rapidly absorbed, with peak brain concentrations achieved 4 h after a single dose, and is present mainly as glucuronide, sulfate, and methylated derivatives. Brain tissue concentrations (28–35 ng·g^−1^) are higher than plasma levels, suggesting selective enrichment (96). Uro-A modulates key anti-aging and autophagy pathways (AMPK, SIRT, mTOR), although its direct molecular targets require further elucidation (97). Urolithin C (Uro-C) has also been shown to reduce Aβ_1–42_ deposition and maintain cholinergic balance in aging models (98).

While preclinical evidence robustly supports the neuroprotective and anti-inflammatory roles of urolithins, human data remain limited and inconsistent. Uro-A is generally recognized as safe (GRAS) by the FDA and exhibits moderate BBB permeability and multi-target mitochondrial benefits (99, 100). However, the absence of large, dose-escalation, and long-term RCTs, particularly those stratified by UM phenotype, is a major gap in clinical translation. Overall, urolithins are promising agents for cognitive protection, but future studies should focus on population stratification and rigorous long-term evaluation.

### 4.2 Sulforaphane: a neuroregulator in the glucosinolate–isothiocyanate pathway

Cruciferous vegetables such as broccoli and kale are rich in glucoraphanin, which is hydrolyzed by plant myrosinase or microbial β-thioglucosidase to produce sulforaphane (SFN). SFN covalently binds cysteine residues in Keap1, leading to Nrf2 nuclear translocation and activation of ARE-dependent antioxidant and detoxification genes. In addition, SFN reversibly inhibits HDAC through thiol alkylation, increasing histone acetylation and upregulating neurotrophic factors such as BDNF (101, 102).

Twelve-week dietary supplementation with SFN in animal models enhances hippocampal PGC-1α, NRF-1, and TFAM transcription, promotes mitochondrial biogenesis, and mitigates age-related cognitive decline (103). SFN also increases BDNF expression and synaptic plasticity through HDAC inhibition in AD mouse and neuron models (102). Early clinical trials are encouraging: a 12-week, double-blind RCT of 30 mg/day SFN improved spatial orientation and working memory in traumatic brain injury patients (104), while similar interventions in older adults improved overall cognitive performance and selectively benefited processing speed and working memory (105, 106).

However, current studies are limited by small sample sizes, lack of dose-gradient design, and short follow-up durations. Long-term safety and the minimum effective dose remain unclear, emphasizing the need for multi-center RCTs with pharmacokinetic monitoring. In summary, SFN demonstrates strong neuroprotective mechanisms across preclinical and early clinical studies, but definitive large-scale evidence is still needed.

### 4.3 Equol: the soy isoflavone–gut microbial estrogen pathway

Daidzein, a major soy isoflavone, is metabolized by gut bacteria such as Slackia isoflavoniconvertens into equol. Only 30–50% of Asian adults are equol producers, underlining the critical influence of the gut microbiota phenotype on efficacy (107, 108). Equol supplementation activates the ERβ-PI3K–Akt signaling pathway and improves spatial memory in animal models (109).

Epidemiological evidence from large Japanese cohorts supports a link between soy isoflavone intake and reduced cognitive impairment risk (110, 111), though most studies do not directly assess equol producer status. Cross-sectional studies show that S-equol producers exhibit better cognitive scores and lower MCI prevalence (112), but findings in other populations (e.g., Singaporean, Chinese, and Asian-American) are inconsistent, likely due to lower isoflavone intake and lack of phenotype assessment (113, 114).

Meta-analyses of RCTs suggest that soy isoflavones provide small improvements in global cognition and memory, but with considerable limitations in sample size and follow-up (115, 116). Mechanistically, equol combines estrogenic, antioxidant, and anti-inflammatory properties, affecting vascular function, metabolic homeostasis, and neuroinflammation—all relevant to the prevention of vascular cognitive impairment (VCID). Future research should stratify participants by equol producer status, use long-term, dose-gradient RCTs, and comprehensively monitor estrogen-related adverse effects. Overall, equol represents a microbiota-dependent, multi-modal neuroprotective agent with particular potential in precision interventions.

### 4.4 Hesperetin/naringenin: citrus flavonoid monomethoxylation products

Citrus flavonoids such as hesperidin and naringin are converted by microbial O-methyltransferase–positive bacteria (e.g., *Clostridium orbiscindens*) into hesperetin and naringenin. These conversions require microbial cleavage of glycosidic bonds and demethylation (117). Alzheimer's disease (AD) features progressive memory decline, Aβ plaque deposition, tau hyperphosphorylation (via GSK-3β activation), and impaired insulin signaling (118–121). In this pathological context, naringenin has shown anti-inflammatory, antioxidant, anti-apoptotic, and neuroprotective effects (122).

In AD models, naringenin improves spatial learning and memory, modulates the PI3K/AKT/GSK-3β pathway, reduces tau phosphorylation, restores insulin signaling and PPAR-γ activity, and confers both metabolic and neuroprotective benefits (123, 124). *In vitro*, naringenin protects against Aβ-induced apoptosis in neuronal cells by regulating caspase-3, PI3K/AKT, and GSK-3β (125). Animal studies show that oral naringenin reduces hippocampal lipid peroxidation, neuronal apoptosis, and reverses memory loss; its neuroprotection is partly estrogen receptor–dependent (126). Naringenin also inhibits AChE and BACE1, restoring memory, and targets multiple AD-related enzyme pathways (127–129).

Collectively, current evidence supports naringenin's multi-target protective actions against AD-related cognitive impairment via the modulation of amyloid/tau pathology, PI3K/AKT/GSK-3β and insulin pathways, cholinergic neurotransmission, the CRMP2 axis, and enzyme inhibition, as well as through antioxidative and anti-inflammatory mechanisms. Its estrogenic and metabolic regulatory properties further reinforce its candidacy as a multi-target nutraceutical-pharmaceutical for future clinical evaluation.

## 5 Conclusion and future perspectives

This review systematically analyzed the complex interplay between dietary phytochemicals and the gut microbiota and how this interaction impacts cognitive health. Based on a comprehensive synthesis of recent research evidence, several key conclusions can be drawn.

Dietary patterns and gut microbial community composition jointly represent major determinants of cognitive function. Through multiple mechanisms, they improve cognitive performance and effectively prevent or delay the onset and progression of age-related neurodegenerative diseases. Phytochemicals in the daily diet play a central role in maintaining gut ecological balance, modulating systemic inflammatory responses, and supporting cognitive health. These bioactive compounds exhibit multidimensional physiological regulatory functions: they synergistically suppress excessive inflammation, promote the development of a diverse and functionally balanced gut microbiota, and establish a robust metabolic signaling network. Collectively, these features constitute the biological foundation for maintaining both gut and cognitive health.

Importantly, there is marked inter-individual variability in the efficiency of microbial metabolite production, largely determined by the unique characteristics of each person's gut microbiota. Evidence indicates that different microbial metabolic phenotypes (e.g., urolithin metabotypes) exert distinct effects on cognitive function, providing an important theoretical basis for the development of personalized nutritional interventions. Bioactive metabolites produced during microbial biotransformation—such as urolithin A, sulforaphane, equol, and hesperetin—generally exhibit higher bioavailability and greater biological activity compared to their parent phytochemicals. These metabolites act as key mediators of the cognitive protective effects associated with plant-based foods.

Crucially, the alleviation of gut dysbiosis and chronic low-grade inflammation—both recognized as central pathological hallmarks of cognitive decline—has emerged as a major strategy for the prevention and intervention of cognitive disorders. By precisely modulating gut–brain axis signaling, plant-derived bioactive compounds optimize neuroimmune function, enhance neural plasticity, and upregulate neurotrophic factor expression, thus providing comprehensive protective mechanisms for cognitive health.

With ongoing advances in precision nutrition and gut microbiome research, the future holds promise for the development of personalized dietary interventions tailored to specific microbial metabolic phenotypes, thereby maximizing the cognitive benefits of phytochemicals. Moreover, integrated studies combining gut microbiome profiling and cognitive function assessment will facilitate a more comprehensive understanding of the diet–microbiota–brain interaction network, supporting the development of more effective and scientifically grounded nutritional strategies for cognitive disorder prevention. At the clinical application level, the development of functional foods enriched in key phytochemicals and specific probiotic formulations may offer new avenues for the protection of cognitive health, particularly for individuals with limited microbial metabolic capacity.
